# Singlet Oxygen Energy for Enhancing Physiological Function and Athletic Performance

**DOI:** 10.3390/bioengineering12020118

**Published:** 2025-01-27

**Authors:** Chia-Feng Hsieh, Chun-Ta Huang, Cheng-Chung Chang, Tun-Pin Hung

**Affiliations:** 1 Graduate Institute of Biomedical Engineering, National Chung Hsing University, Taichung 402, Taiwan; foolish5021@gmail.com; 2 Protrustech Co., Ltd., 3F.-1, No. 293, Sec. 3, Dongmen Rd. East District, Tainan City 701, Taiwan; win@protrustech.com; 3 Office of Physical Education, Tamkang University, New Taipei City 251301, Taiwan

**Keywords:** singlet oxygen energy generator, photosensitizer, singlet oxygen, singlet oxygen energy, lactate

## Abstract

A total of 75% of the oxygen humans inhale is exhaled without being utilized. To help organisms better utilize oxygen in exercise training, we designed the singlet oxygen energy generator (SOEG), a device that converts ambient air into energy-rich oxygen. The SOEG comprises an LED light source, a photosensitizer kit, and an air pump. Based on the principle of photosynthesis, the photosensitizer activates oxygen to produce excited-state singlet oxygen under the irradiation of light, which releases about 94 kJ/mol of singlet oxygen energy (SOE) after the relaxation process. After comparing data from 14 volunteers, we found that inhaling SOE during exercise significantly reduces energy consumption during running, decreases oxygen uptake, and improves running efficiency. At the same time, SOE effectively lowers blood lactate levels and improves oxygen utilization, indicating that SOE may enhance endurance and efficiency during exercise.

## 1. Introduction

Oxygen is an abundant and multifaceted element. The most common and consequential form is molecular oxygen (O_2_), a diatomic molecule indispensable for aerobic cellular metabolism. However, oxygen also exists in more reactive forms, including its free radical derivatives, which are highly unstable. Another form is produced when the ground-state oxygen molecule O_2_ is excited to a higher energy state, forming singlet oxygen (SO), a species with paired electrons. This form of oxygen is considered harmful within biological systems [1]. Singlet oxygen exists in trace amounts in the upper atmosphere, often generated by the excitation energy from sunlight. Solar radiation can be directly absorbed by ground-state triplet oxygen (^3^O_2_), leading to the excitation and formation of singlet oxygen (^1^O_2_) [2]. Additionally, biological reactions involving ozone, proteins, methionine, thiols, ascorbic acid, and triphenyl phosphate can contribute to the generation of singlet oxygen in the atmosphere [3,4,5].

**Figure 1:**
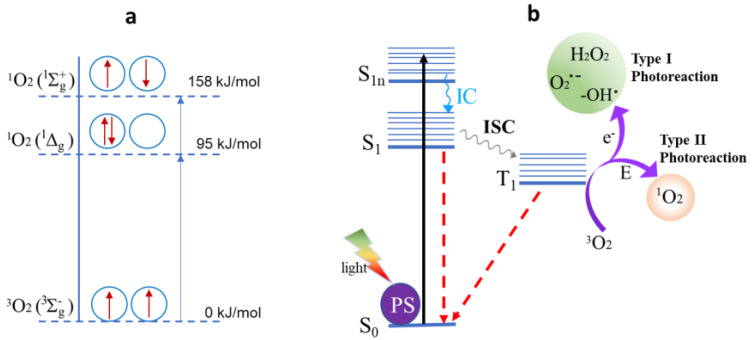
(**a**) The energy difference between singlet oxygen in its excited state and triplet oxygen in its ground state. (**b**) How light excites a photosensitizer, leading to the generation of singlet oxygen. The e^-^ and E in the arrows represent electron transfer and energy release pathways, respectively.

In other words, singlet oxygen represents a form of reactive oxygen species (ROS) resulting from the “activation” of triplet oxygen. As shown in Figure 1a, the ground state of oxygen is a triplet (with two parallel unpaired electrons in its outer orbitals), while the two higher energy states, ^1^Δg and ^1^Σg^+^, have paired electrons with parallel and antiparallel spins, respectively. The former, ^1^Δg, is a singlet electronic state with approximately 94 kJ/mol of excess energy relative to ground-state molecular oxygen, making it kinetically unstable under ambient conditions and thus a type of ROS [6,7,8]. This reactivity is implicated in various toxic mechanisms within biological systems.

Although singlet oxygen is not a free radical, it possesses significant energy to “attack” other substances. This non-ionic and non-radical form of oxygen can rapidly diffuse in both air and water. That is why singlet oxygen is considered a toxin in both the natural and biological environments. However, its high reactivity can also be harnessed for new scientific applications. For example, singlet oxygen can induce the oxidation of electron-rich hydrocarbons without requiring high temperatures, enabling new applications in (bio)chemical reactions and combustion processes [6,7,9]. Additionally, it is applied in water purification and wastewater treatment [10], lipid peroxidation [11,12], and the photodegradation of polymers for drug delivery [13]. Given these properties, highly reactive oxygen species have been explored as a key to technological breakthroughs and have driven advancements in environmental, biotechnological, materials, medical, and defense sciences. A well-known application is photodynamic therapy (PDT) for cancer treatment [14,15].

**Figure 2:**
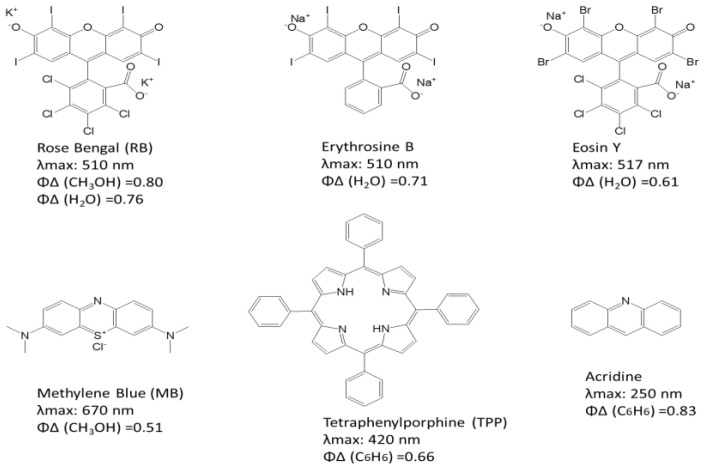
Typical photosensitizers and their related singlet oxygen yield in solvents [19].

How is singlet oxygen produced? Given its highly reactive nature, it can be artificially generated through methods such as discharge machining [16] or via surface-mediated reactions on particles such as silicon dioxide, aluminum oxide, and transition metal oxides [17]. The abundant oxygen, light, and natural photosensitizers (PSs, such as chlorophyll) combine through photosynthesis, the natural way to produce singlet oxygen, as shown in the production mechanism in Figure 1b. Hence, the photosynthesis principle became the design template for artificially producing singlet oxygen [18]. This involves techniques such as photosensitization and photochemical reactions. Choosing an appropriate photosensitizer is crucial for efficient singlet oxygen generation. The properties of such a photosensitizer should include a high light absorption at the wavelength of study (i.e., a high extinction coefficient of light), a high quantum yield Φ_Δ_, and resistance to oxidation by singlet oxygen or other oxidants present in the system (i.e., a long lifetime for the excited triplet state). Some typical dyes and their structures, absorbance range, and quantum yield in different solutions are illustrated in Figure 2 [19].

Even though singlet oxygen is known to be toxic in biological systems, the energy it releases may be beneficial. As an excited-state product, singlet oxygen is highly reactive and can attack targets indiscriminately by releasing energy to return to the ground state. When this occurs in the body, it can cause harm. However, if there is no “target” to attack, the energy of singlet oxygen will return to the ground state and release 94 kJ/mol of energy (equivalent to 22.4 kcal/mol), [20] called singlet oxygen energy (SOE), which is relatively beneficial energy absorbed by biological organisms [21]. The theoretical basis of our study is as follows:

- We deliberately produced a large amount of SO with no targets to attack, preventing energy transfer. Eventually, the excited singlet oxygen returned to its ground state by releasing 94 kJ/mol of energy, which supported the potential for novel applications and generated unexpected results [22].
- According to the principle of antibodies, when there are few harmful substances invading the human body, the defense capabilities of the immune system can be stimulated. Therefore, a small amount of ROS is indeed beneficial to the body [23].
- A total of 75% of the oxygen humans inhale is exhaled without being utilized. The key point is therefore not the supply or concentration of oxygen in the air but how efficiently the body uses the available oxygen for energy metabolism. That is, instead of the conventional method of increasing oxygen supply, we propose a novel approach to enhancing the energy of the existing oxygen.

**Figure 3:**
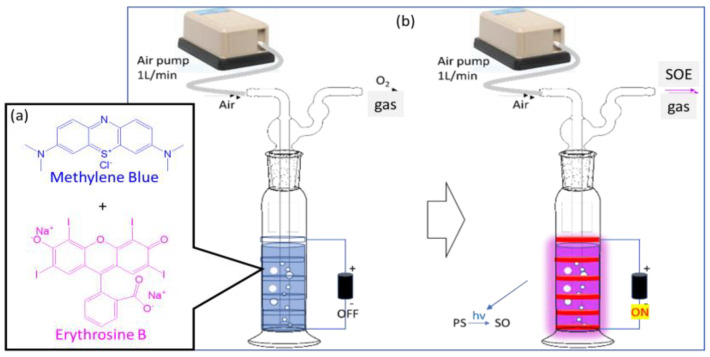
Design of the singlet oxygen energy generator (SOEG): (**a**) photosensitizer-containing solution (methylene + erythrosine B). (**b**) Construction of the SOEG: photosensitizer solution, LED, and pump.

**The purpose of our research can be summarized as follows:** Previous studies have explored the effects of singlet oxygen energy (SOE) on exercise performance and demonstrated that SOE can enhance exercise performance by increasing endurance and energy utilization, reducing heart rate, improving oxygen consumption, and decreasing carbon dioxide production [24,25,26]. Additionally, studies have shown that singlet oxygen energy can reduce blood lactate levels and enhance antioxidant activity [27,28,29]. However, these studies have primarily relied on animal models or involved pre-exercise SOE inhalation without continuous exposure during exercise. Consequently, there is a paucity of research investigating the impact of a sustained SOE-rich environment on exercise performance. The present study aimed to address this gap by exposing participants to a SOE-rich environment both before and throughout the exercise to identify environmental factors that may enhance endurance. To this end, we designed a singlet oxygen energy generation (SOEG) device, as shown in Figure 3. In the SOEG, normal ambient air passes through a photosynthesis-like system to excite the oxygen in the air to a higher energy state, releasing energy and combining it with water molecules. This device employs a novel approach, utilizing a photosensitive solution kit (photosensitizer in Figure 2) and LED illumination, in contrast to the arc-based methods used by other devices [25,26]. This approach significantly reduces the cost of the device. In this study, healthy individuals inhale SOE, and relevant fitness data are tested.

## 2. Materials and Methods

### 2.1. Photosensitive Solution Kit

The photosensitive material employed in this study is a mixture of methylene blue (MB) and erythrosine B (EB) in aqueous solution, as depicted in Figure 2. MB, a salt commonly used as a dye and medication [30], is listed on the World Health Organization’s list of essential medicines [31]. EB, also known as FD&C Red No. 3 [32], is an organoiodine compound derived from fluorone and is primarily used as a food coloring [33]. For this study, we prepared a photosensitive solution kit containing 10 µM MB (120 mL) and 10 µM EB (180 mL) in a total volume of 300 mL, as illustrated in Figure 3a.

### 2.2. Design of SOEG Device

We designed the singlet oxygen energy generator (SOEG) to help organisms better utilize the oxygen in inhaled air. This instrument passes normal ambient air through a photosynthesis-like system to excite the oxygen in the air to a higher energy state, releasing SOE and combining it with water molecules. As shown in Figure 3b, the device incorporates an air pump (air output 2000 cc/min; pressure 0.25 kg/cm^2^; power consumption 5 W; dimensions: 155 × 92 × 60 mm; weight 625 g; power cord 3 feet) and an LED flexible light strip (LED 5050 SMD*60 beads/m; input voltage: DC12V; operating current 0.6 A/m; output power: 7.2 W/m) to activate the photosensitizer solution kit (300 mL of a 10 µM MB and EB aqueous solution) and achieve optimal performance. For safety reasons, the SOEG was labeled as follows: “Do not expose to liquids, especially when system is operating (during irradiation)”.

### 2.3. Instrumentation for Analysis

#### 2.3.1. Gas Analysis System

The MetaMax 3B (Cortex Biophysik GmbH, Leipzig, Germany) is a mobile spiroergometer employed for cardiopulmonary exercise testing that enables precise measurement of oxygen uptake (VO_2_) and carbon dioxide production (VCO_2_).

#### 2.3.2. Treadmill

The Trackmaster TMX425C (Davis Medical Electronics, Cades Way, Vista, CA, USA) is a high-precision laboratory treadmill featuring a speed range of 8–19 km/h, a zero-start option, and an elevation range of 0–25%.

#### 2.3.3. Heart Rate Sensor

The T31 Coded™ transmitter was used to measure heart rate status.

#### 2.3.4. Blood Lactate Testing

The Lactate Pro-LT-1730 from (Arkray, Minneapolis, MN, USA) was employed for rapid and accurate measurement of blood lactate levels. This device provides precise results, making it suitable for monitoring lactate concentrations during exercise testing and physiological assessments.

### 2.4. Methods

#### 2.4.1. Experimental Tests

A pilot study was conducted with 14 participants, including 6 females (average age: 24.2 years; BMI: 19.7) and 8 males (average age: 24.3 years; BMI: 22.8). The participants underwent two tests separated by a 4-day recovery period. In the first test, they breathed air from the SOEG device (clean aqueous air without the photosensitizer solution kit) for 50 min, followed by a 10 min rest before undertaking exercise tests. The same procedure was followed in the second test with the SOEG device (with the photosensitizer solution kit to generate SOE). All the participants were blinded to the conditions. There was a four-day rest period between the two tests, during which the participants followed the following protocol: avoiding any other strenuous or sweaty exercise, maintaining a normal diet, and avoiding staying up late. The indoor test environment was temperature-controlled at 24 °C (standard conditions), especially for heart rate measurements taken at different stages of exercise.

Participants were exposed to a higher energy environment created by the SOEG equipped with SOE. After this pre-exercise period (held in a standard temperate environment of 20–22 °C for 60 min before exercise), the participants underwent a treadmill test while remaining in the enhanced environment. Their respiratory gases and heart rate were monitored using the same MetaMax 3B and Polar T31 devices, with data also collected every 10 min during the exercise. After the exercise test, the participants were asked to rest in the lounge. The research team confirmed that the subjects had recovered before asking them to fill in the questionnaire (ex: RPE).

#### 2.4.2. Submaximal Running Exercise Test

The submaximal treadmill test was designed to assess cardiovascular response and endurance under progressively increasing workloads. The protocol consisted of four stages, each lasting 4 min, resulting in a total test duration of 16 min. The specific procedures were as follows:

1. Initial speed estimation: The starting running speed was determined based on each participant’s usual running pace to ensure comfort and familiarity.
2. Gradual speed increase: At the beginning of each subsequent stage, the treadmill speed was increased by 1 to 1.5 km/h to progressively increase exercise intensity.
3. Rating of perceived exertion (RPE): At the end of the fourth minute of each stage, participants were asked to report their RPE using the Borg scale, as shown in Table 1. The RPE indicates perceived exertion and overall fatigue throughout the test.
4. Physiological measurements: Throughout the test, key respiratory and metabolic parameters were continuously monitored using the MetaMax 3B gas analysis system.

The parameters measured included oxygen uptake (VO_2_), which indicates the body’s efficiency in utilizing oxygen during exercise; respiratory exchange ratio (RER), the ratio of carbon dioxide production to oxygen consumption, reflecting substrate utilization; and ventilation (VE), the volume of air inhaled or exhaled per minute, indicating the respiratory response to exercise intensity.

#### 2.4.3. Maximal Oxygen Uptake (VO_2_ Max) Test

After completing the submaximal running test, participants rested and hydrated until they felt ready to resume running. They then stepped onto the treadmill, re-applied the mask, and reconnected the breathing tube. During this phase, the running speed was fixed based on the participants’ reported RPE of 11–13 from the submaximal test. The incline was gradually increased to 3.5%, 6%, 8.5%, and 11%, with each stage lasting 3 min. At the end of each 3 min stage, participants were asked to report their RPE. Eventually, the highest recorded oxygen uptake during the test was considered the maximum oxygen uptake (VO_2_ max).

#### 2.4.4. Singlet Oxygen Detection

DPBF (1,3-diphenylisobenzofuran), a widely employed reactive oxygen species (ROS) indicator, is an indicator of singlet oxygen. Dimethyl sulfoxide (DMSO) is a powerful solvent, capable of dissolving a wide range of substances, including both polar and nonpolar compounds. In most cases, DMSO is used as a solvent for preparing stock solutions. DPBF was introduced to a DMSO solution containing the photosensitizers in 2.1, and its concentration was monitored under light irradiation conditions. We used a 20 W xenon lamp as a light source, a cube with a 400–700 nm wavelength combined with a 510 nm long pass (LP) filter. Eventually, the light intensity incident on the DMSO solution during the irradiation experiment was measured photometrically as 8 J/cm² (480 W/cm^2^ in min). The 510 LP filter prevented the DPBF from degradation by exposure to ultraviolet light. The DPBF in the mixture solution was easily oxidized by singlet oxygen to evaluate the singlet oxygen activity of its molecules. Therefore, a quartz tube containing 2 mL of molecules and DPBF in DMSO solution placed on an iron stand 5 cm away from the light source was irradiated. The experimental setup was housed in a dark space to prevent the exposure of the molecules and DPBF to light from external light sources. Next, we used an ultraviolet–visible light absorption spectrometer (UV-vis) to measure the absorbance value after irradiation at different times.

## 3. Results

### 3.1. Detection and Analysis of Singlet Oxygen Generation by Photosensitizer Solution Kit

As described in the literature [34], the generation of singlet oxygen can be investigated using DPBF (1,3-diphenylisobenzofuran). In the present study, the characteristic absorption peak of DPBF is situated at 418 nm. Upon interaction with singlet oxygen, the molecular structure of DPBF disintegrates, leading to a gradual decrease in the absorption value at 418 nm as the reaction progresses. Consequently, the generation of singlet oxygen can be directly monitored through changes in the absorption spectrum.

**Figure 4:**
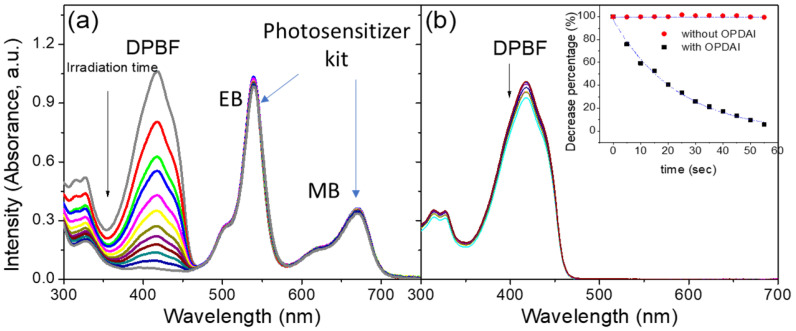
Detection 30 μM of DPBF spectral changes in singlet oxygen at different periods of light exposure. The figure shows the results for systems containing 10 μM of the photosensitive material (**a**) and control systems (without photosensitizer) (**b**). The detection of singlet oxygen was carried out using DPBF.

DPBF was introduced to a DMSO solution containing a photosensitizer solution kit, as prepared in Section 2.1, and its concentration was monitored under light irradiation conditions. Figure 4 shows the absorption spectra changes in the kit mixed with DPBF (Figure 4a) and pure DPBF (Figure 4b) under light exposure. Observing the spectrum variation, the absorption peak of DPBF at 418 nm decreases with increasing light exposure time, indicating that DPBF was gradually decomposing, which suggests a continuous production of singlet oxygen by the photosensitizer kit. Compared with the control group, where no photosensitizer was used, the absorption value of pure DPBF remained unchanged. Thus, it can be inferred that the photosensitizer solution kit indeed produces singlet oxygen when exposed to light, classifying it as a type II ROS photosensitizer and confirming its photosensitizing effect.

### 3.2. Comparative Analysis of Exercise Data with and Without the SOEG System

**Figure 5:**
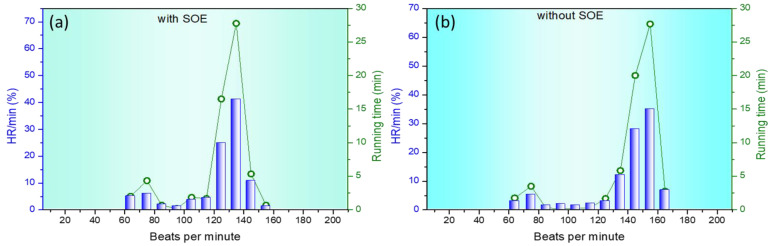
Heart rate relative to overall running performance. Group using SOEG for inhaling SOE for one hour (50 min, resting 10 min) before training (**a)**; control group without SOE (**b**).

Normally, 12.1% of the recorded heart rate values were between 130 and 140, 28.0% between 140 and 150, 35.0% between 150 and 160, and 6.8% were above 160 when the measurements were taken during pre-training air-breathing sessions, as shown in Figure 5b. Compared to the values obtained when using the breathing system to inhale SOE produced by SOEG, heart rates significantly decreased, with 24.8% of the recorded values between 120 and 130, 41.1% between 130 and 140, 10.7% between 140 and 150, and only 1.3% above 150. The results in Figure 5a are very positive, confirming that pre-training use of SOE seems to make it easier for individuals to exert their strength.

Next, we conducted a submaximal running exercise test, with the intensity set at 60%, 70%, 80%, and 90% of the participants’ maximum oxygen uptake (VO_2_max) based on their fitness levels [35]. The submaximal running test consisted of four stages with increasing speeds, based on the participants’ usual running paces, with each stage increasing by 1 to 1.5 km/h. Heart rate, blood lactate, and oxygen uptake were monitored during each stage. Here, “pre-exercise” refers to data collected before exercise, specifically the average values from the last 10 min before the exercise session, and used to examine the effect of the SOE environment on running economy. In this experiment, the average VO_2_max of the 14 participants was 48.2 mL/kg/min.

**Figure 6:**
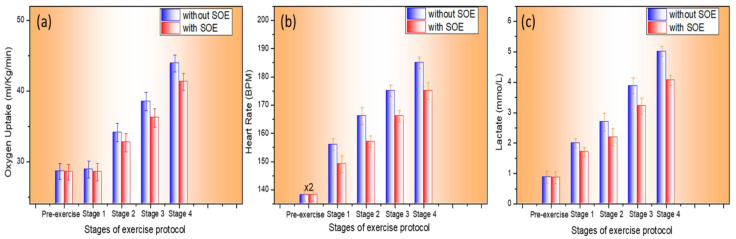
Bar chart comparison of oxygen consumption (**a**), heart rate (**b**), and blood lactate levels (**c**) across four exercise stages between the group that inhaled SOE (red) and the control group without SOE (blue).

As shown in Figure 6a, changes in oxygen uptake during the first stage did not show significant differences when using SOE. However, the difference became significant in the second stage. Significant differences were observed at the third- and fourth-stage speeds; the data indicated that oxygen uptake was significantly lower in the SOE environment compared to the normal environment group. As shown in Table 2, the results indicated that during the submaximal running test, oxygen uptake at each speed stage in the SOE environment was lower than in the control environment. There was no significant difference at the initial speed, but the difference became more significant with increasing speed, eventually reaching a stable difference of approximately 6%. These results provided a foundation for exploring the data in Figure 5 and determining how the SOEG contributes to the observed reduction in exercise heart rate.

As depicted in Figure 6b, we hypothesized that using the SOEG and inhaling SOE would reduce the heart rate during exercise, particularly during strenuous exercise. Table 2 illustrates that once a certain percentage of heart rate improvement is achieved, the value tends to plateau as a steady state (approximately 5% in this experiment). This may be related to the amount and duration of SOE inhalation, which will be further studied. Additionally, Figure 6c reveals that the inhalation of SOE results in a decrease in blood lactate levels during exercise, especially during the third and fourth stages. Similarly, the data reach a plateau at a certain percentage (approximately 12% in this experiment), as shown in Table 2. In summary, the results in Figure 6 indicate that oxygen uptake ratios decreased by about 6% at different stages of exercise, blood lactate levels dropped by approximately 12%, and recorded heart rates showed a maximum decrease of 5%. These results also confirm the participants’ own impressions (from the questionnaire in Section 2.4.1), with many reporting significant and positive improvements in their performance after using the SOEG.

## 4. Discussion

**Safety issues:** Singlet oxygen exhibits a short lifetime in water, typically in the microsecond range (3.1 µs in pure water, 68 µs in D_2_O), due to quenching by water molecules and solutes. In air, its lifetime can be longer (~54 ms) but is significantly reduced by impurities or quenching agents [36]. Our device is specifically designed to ensure that the output gas from the SOEG does not contain singlet oxygen. Instead, participants inhale the energy released (SOE) during the relaxation process of the excited singlet oxygen.

**Aerobic and anaerobic exercise:** Previous studies have shown that singlet oxygen energy can enhance endurance and energy utilization during aerobic exercise while simultaneously reducing heart rate, increasing oxygen consumption, decreasing carbon dioxide production, and augmenting energy production [25,26,27]. A decrease in inspired oxygen concentration can impair performance during endurance exercise, primarily due to insufficient oxygen supply. This lack reduces the utilization of aerobic pathways and promotes a greater reliance on the anaerobic metabolism, ultimately leading to muscle fatigue and compromised performance. During long-duration submaximal exercise, glucose and fatty acids serve as the primary energy sources, and oxygen is required in energy production. Aerobic training increases oxygen availability; therefore, hypoxia, lack of oxygen utilization, and insufficient blood flow are key factors that contribute to muscle fatigue.

When anaerobic glycolysis becomes the primary energy source, excessive lactic acid production occurs, forcing the athlete to either stop or slow down. This is particularly pronounced in untrained individuals, where lactate accumulation often leads to muscle stiffness and discomfort. For endurance athletes, the ability to clear lactate is crucial for optimal performance. This study found that SOE intervention can effectively reduce blood lactate levels, thereby improving oxygen utilization. Given that lactate clearance or metabolism requires sufficient oxygen, it is reasonable to infer that singlet oxygen energy may enhance lactate clearance or contribute to energy metabolism. Based on the oxygen uptake measurements from the submaximal running tests, energy consumption during running significantly decreased with SOE intervention, which caused a significant decrease in oxygen uptake and a significant improvement in running economy. In other words, with SOE intervention, the body’s oxygen utilization becomes more efficient during submaximal running, enhancing the efficiency of oxygen use.

Nevertheless, there remain many aspects of SOE that warrant discussion and clarification. The current study limitations and suggested remedies include the following: (1) Sample size limitation—future studies should increase the sample size to improve data certainty. (2) Sample type limitation—we should investigate and compare the benefits of SOE between trained individuals (such as professional athletes) and untrained individuals. We should also examine the differences in SOE benefits between the elderly and young people to explore the applications in long-term elderly care. (3) Study design limitation—in this study, we administered SOE before exercise; we should also investigate the differences between administering SOE during and after exercise. (4) Methodological limitation—how does SOE affect daily life without exercise, especially for elderly people and recovering patients? The results could expose the issues related to randomized treatment sequences.

## 5. Conclusions

Efficient energy utilization is paramount in endurance exercises. This study investigated the impact of singlet oxygen energy (SOE) on running economy. Human trials demonstrated that employing the SOE breathing technique to reduce oxygen uptake, heart rate, and blood lactate levels by 6%, 5%, and 12%, respectively, compared to the control group. These results indicate that energy expenditure during submaximal running was lower when using the SOEG, suggesting that training in an SOE-enriched environment may optimize energy efficiency and create a better running economy. Endurance exercises, whether in training or competition, demand prolonged physical exertion. From the perspective of energy consumption, SOE can be provided during training to improve training quality. Future research will explore the optimal timing of SOEG use and its potential applications during exercise. In conclusion, while oxygen is essential for humans, it can become reactive oxygen species (ROS) under external influences such as light or chemical reactions. However, ROS are not solely harmful agents. As demonstrated in this study, when used appropriately, ROS, particularly when generated by SOE, may also be beneficial to the human body.

## Figures and Tables

**Table 1:** Rating of perceived exertion (RPE) items used during the submaximal running test.

Stage	Duration (Minutes)	Speed Increase (km/h)	RPE Assessment Time
**1**	0–4	Baseline	4th min
**2**	4–8	+1.0 to 1.5	8th min
**3**	4–8	+1.0 to 1.5	12th min
**4**	12–16	+1.0 to 1.5	16th min

**Table 2:** Comparison of oxygen uptake, heart rate, and blood lactate levels across four exercise stages between the group inhaling SOE and the control group.

	Oxygen Uptake (ml/Kg/min)			Heart Rate (Beats/min)			Lactate (mmol/L)		
	Without SOE	With SOE	Improve ∆ (%)	Without SOE	With SOE	Improve ∆ (%)	Without SOE	With SOE	Improve ∆ (%)
**Pre-exercise**	28.6 ± 1.1	28.5 ± 1.4	-	69 ± 2	69 ± 2	-	0.88 ± 0.15	0.86 ± 0.1	-
**Stage1**	28.9 ± 1.2	28.5 ± 1.1	1.38	156 ± 2	149 ± 3	4.49	1.99 ± 0.14	1.80 ± 0.2	9.55
**Stage2**	34.1 ± 1.3	32.2 ± 1.3	4.10	166 ± 3	157 ± 2	5.42	2.69 ± 0.29	2.35 ± 0.2	12.63
**Stage3**	38.5 ± 1.3	36.2 ± 1.5	5.97	175 ± 2	166 ± 2	5.14	3.88 ± 0.26	3.41 ± 0.3	12.11
**Stage4**	43.9 ± 1.2	41.3 ± 1.6	5.92	185 ± 2	175 ± 3	5.41	5.01 ± 0.29	4.36 ± 0.2	12.97

## Data Availability

Data available in a publicly accessible repository.
